# Comparative modeling and mutual docking of structurally uncharacterized heat shock protein 70 and heat shock factor-1 proteins in water buffalo

**DOI:** 10.14202/vetworld.2019.2036-2045

**Published:** 2019-12-23

**Authors:** Ravinder Singh, Ankita Gurao, C. Rajesh, S. K. Mishra, Saroj Rani, Ankita Behl, Vikash Kumar, R. S. Kataria

**Affiliations:** 1 ICAR-National Bureau of Animal Genetic Resources, Karnal, Haryana, India; 2 Department of Biotechnology, Sri Guru Granth Sahib World University, Fatehgarh Sahib, Punjab, India; 3 Department of Veterinary Microbiology and Biotechnology, Rajasthan University of Veterinary and Animal Sciences, Bikaner, Rajasthan, India; 4 Department of Agriculture, Maharishi Markandeshwar University, Ambala, Haryana, India; 5 Department of Biotechnology, Guru Nanak Dev University, Amritsar, Punjab, India; 6 Department of Molecular Biology and Biochemistry, Guru Nanak Dev University, Amritsar, Punjab, India

**Keywords:** *Bubalus bubalis*, docking, heat shock proteins, heat shock factor-1, heat shock protein 70, homology modeling

## Abstract

**Aim:**

In this study, a wide range of *in silico* investigation of *Bubalus bubalis* (BB) heat shock protein 70 (HSP70) and heat shock factor-1 (HSF1) has been performed, ranging from sequence evaluation among species to homology modeling along with their docking studies to decipher the interacting residues of both molecules.

**Materials and Methods:**

Protein sequences of BB HSP70 and HSF1 were retrieved from NCBI database in FASTA format. Primary and secondary structure prediction were computed using Expasy ProtParam server and Phyre2 server, respectively. TMHMM server was used to identify the transmembrane regions in HSP70. Multiple sequence alignment and comparative analysis of the protein was carried out using MAFFT and visualization was created using ESPript 3.0. Phylogenetic analysis was accomplished by COBALT. Interactions of HSP70 with other proteins were studied using STRING database. Modeller 9.18, RaptorX, Swiss-Modeller, Phyre2, and I-TASSER were utilized to design the three-dimensional structure of these proteins followed by refinement; energy minimization was accomplished using ModRefiner and SPDBV program. Stereochemical quality along with the accuracy of the predicted models and their visualization was observed by PROCHECK program of PDBsum and UCSF Chimera, respectively. ClusPro 2.0 server was accessed for the docking of the receptor protein with the ligand.

**Results:**

The lower value of Grand Average of Hydropathy indicates the more hydrophilic nature of HSP70 protein. Value of the instability index (II) classified the protein as stable. No transmembrane region was reported for HSP70 by TMHMM server. Phylogenetic analysis based on multiple sequence alignments (MSAs) by COBALT indicated more evolutionarily closeness of *Bos indicus* (BI) with *Bos taurus* as compared to BI and BB. STRING database clearly indicates the HSF1 as one of the interacting molecules among 10 interacting partners with HSP 70. The best hit of 3D model of HSP70 protein and HSF1 was retrieved from I-TASSER and Phyre2, respectively. Interacting residues and type of bonding between both the molecules which were docked by ClusPro 2.0 were decoded by PIC server. Hydrophobic interactions, protein-protein main-chain-side-chain hydrogen bonds, and protein-protein side-chain-side-chain hydrogen bonds were delineated in this study.

**Conclusion:**

This is the first-ever study on *in silico* interaction of HSP70 and HSF1 proteins in BB. Several bioinformatics web tools were utilized to study secondary structure along with comparative modeling, physicochemical properties, and protein-protein interaction. The various interacting amino acid residues of both proteins have been indicated in this study.

## Introduction

Environmental stresses instigate physiological responses in living organisms as part of their adaptation mechanism. Temperature deflections produce a heat shock response in living organisms, which comprise the expression of heat shock proteins (HSPs). Synonymously known as molecular chaperones, these proteins are in charge of regulating proteostasis, whenever any kind of physical or chemical trigger disturbs the internal milieu of the cellular organization [1-4]. Among this huge family of proteins, HSP70 is one of the members with a molecular weight of 70 kDa, comprising various isoforms. Rising levels of HSPs have been reported throughout the exposure to diverse environmental stresses, including heat and HSP70 to function as an indicator of thermotolerance in cells [5]. The expression of the HSP-encoding genes is mainly under the transcriptional control of heat shock factor-1 (HSF1) [6-11]. HSF1 is a monomer, which complexes with HSP70 during an unstressed condition and the complex disintegrates when the stressors come into action, resulting in trimerization of HSF1 monomers. This leads to the activation of HSF1 followed by transcription of HSP70 gene [12]. This is a ubiquitous mechanism owned by all mammalian systems.

In the present time, the ever-changing global climatic conditions have made abiotic stresses such as heat, a major challenge for productivity in livestock [13,14]. In general, animals require an ambient temperature, which is crucial for proper physiological functioning, referred to as the thermal comfort zone [15]. Heat stress is a consequence of exposure of the livestock to a temperature more than the upper critical temperature of the range. Animals have developed phenotypic responses to various sources of stress such as heat called acclimatization [16]. Acclimatization results in reduced feed intake, increased water intake, and altered physiological functions such as reproductive and productive efficiency and a change in respiration rate [17]. Buffalo (*Bubalus bubalis* [BB]) in India, is an important livestock species among all domestic animals because it alone contributes to the highest to the country’s milk as well as meat production [18,19]. However, the black skin coat color of buffalo proves as a major drawback. These challenges are counterfeited by some special morphological characters which include the protection from ultraviolet due to the excess melanin secretion by the black skin coat, aid in reflecting the solar radiations [20]. As the heat stress has a systemic impact on the biological system, the morphological features may be helpful to some extent in aiding buffaloes for better physiological comfort but the parallel responses taking place due to heat stress at the cellular level, are more deleterious. The cellular consequences of heat stress include protein unfolding, entanglement, unspecific aggregation, and increase in membrane fluidity [21,22]. This is where HSPs, especially HSP70, which is abundant comes into action [5]. As a 70-kDa HSP, HSP70 facilitates a stunning array of diverse functions [23,24]. HSP70 assists a wide range of folding processes, including the folding and assembly of newly synthesized proteins, refolding of misfolded and aggregated proteins, membrane translocation of organelle and secretory proteins, and control of the activity of regulatory proteins [25-29]. The availability of preliminary structural data is, therefore, required for exploring possible imputes of HSP70 in BB due to morphologically divergent acclimatization to that of cellular components. Furthermore, the HSP70 shares a high percent of similarity among various eukaryotes, which makes it an approachable candidate to evaluate functional roles using *in silico* studies.

To date, there is no availability of experimental structure of HSP70 protein for BB, and therefore, evaluation of HSP70 in BB becomes essential to analyze the prospective mechanism of its functioning in buffalo and interaction studies with other molecules. The study has also focused on finding the other interacting partners of HSP70 protein and also to deduce their roles during cell cycle regulation and signal transduction in BB, utilizing various *in silico* tools.

## Materials and Methods

### Ethical approval

The present work did not involve any wet laboratory experiments. However, all the animal handling procedures followed under the project had the Institute Animal Ethics Committee approval vide sanction F.No. NBAGR/IAEC/2017 dated 21-01-2017.

### Sequence extraction

The primary sequence of the HSP70 (Accession No. ADQ27308.1) and HSF1 (Accession No. AHB38918.1) of BB was extracted from the reference protein database of NCBI. Both the protein sequences were retrieved in FASTA format and used for further analysis.

### Primary and secondary structure prediction

For HSP70 and HSF1 proteins’, primary structure prediction, molecular weight, theoretical isoelectric point (pI), total number of positive and negative residues, extinction coefficient [30], instability index [31], aliphatic index [32], and grand average of hydropathicity (GRAVY) [33] were computed using the Expasy ProtParam server (https://web.expasy.org/protparam/). Secondary structure of this protein was predicted using the FASTA sequences of HSP70 and HSF1 using Phyre2 server [34].

### Comparative sequence analysis of HSP70 protein

The homologous HSP70 sequences of 10 species, i.e., BB, *Bos indicus* (BI), *Bos taurus* (BT), *Capra hircus* (CH), *Ovis aries* (OA), *Rattus norvegicus* (RN), *Sus scrofa* (SS), *Equus caballus* (EC), *Mus musculus* (MM), and *Homo sapiens* (HS), were fetched from protein reference database of NCBI (Table-1). MSA was done using MAFFT MSA tools and visualization was carried out using ESPript 3.0 [35,36], which divulge crucial dissimilitude in BB HSP70 protein as compared to other species. Phylogenetic analysis was accomplished using COBALT [37].

**Table-1:** Accession numbers used for multiple sequence alignment of HSP70 from different species.

Species	Accession number
*Bubalus bubalis*	ADQ27308.1
*Bos indicus*	AEX55799.1
*Bos taurus*	AAA73914.1
*Capra hircus*	NP_001272632.1
*Ovis aries*	NP_001254803.1
*Rattus norvegicus*	NP_001316825.1
*Sus scrofa*	P34930.1
*Equus caballus*	NP_001243852.1
*Mus musculus*	NP_034609.2
*Homo sapiens*	AAD21816.1

### Annotation of interacting partners of HSP70

STRING database (https://string-db.org/) was utilized to know the critical interactions displayed by HSP70 in BB for performing diverse cellular activities, and data on protein-protein interactions were collected [38].

### Model building, evaluation, and docking studies of HSP70 and HSF1

The modeling of the three-dimensional structure of these proteins was constructed by various tools and servers such as Modeller 9.18, RaptorX, Swiss-Modeller, Phyre2, and I-TASSER; the best hit of HSP70 protein was obtained from I-TASSER (https://zhanglab.ccmb.med.umich.edu/I-TASSER/) and the best hit of 3D model of HSF1 was generated by Phyre2. After model construction using I-TASSER and Phyre2 (http://www.sbg.bio.ic.ac.uk/phyre2/) server, the models were subjected to refinement and energy minimization using ModRefiner and Swiss-PdbViewer (SPDBV) program [39,40]. PROCHECK program of PDBsum and verifies 3D score from SAVES server was used to check the stereochemical quality and precision of the predicted model [41,42]. Finally, the protein was visualized in UCSF Chimera [43].

For the docking studies, HSF1 was docked within the HSP70 homology model using an automated server, ClusPro 2.0, in which receptor was HSP70 protein and HSF1 was used as a ligand. On the basis of different desolvation and electrostatic potential, ClusPro 2.0 can differentiate thousands of conformations of the protein. The generated conformations can be further categorized through clustering and therefore interpreted the most fit structure, normally, the structure found to be closest to native structure from X-ray crystallography results is considered [44,45].

## Results and Discussion

Among various chaperones, HSP70 is one of the major *de novo* protein folding chaperones. The extensive range of cellular functions in eukaryotic organelles is coordinated by HSP70, including refolding, or degradation of misfolded proteins and folding of *de novo* synthesized polypeptides [46]. In brief, HSP70 is a nanomachine that plays a significant part in sustaining the homeostasis and proteostasis of any organism [47]. For farm animals, as HSP70 was identified to be the ideal biological marker for heat stress; therefore, a wide range *in silico* study along with evaluation of structure elucidation and stereochemical properties was performed for buffalo’s HSP70. In addition, the protein-protein interaction of HSP70 and HSF1 was also studied.

### Primary and secondary structure prediction

**Figure-1:**
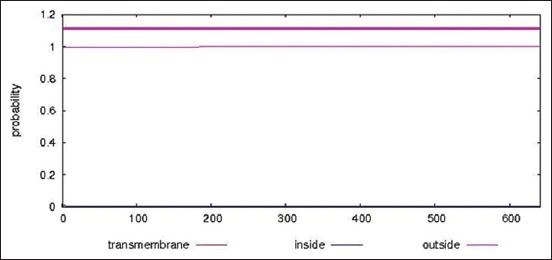
The TMHMM server prediction for the membrane topology of heat shock protein 70.

The primary structure of HSP70 was estimated using Expasy ProtParam server. The server estimated the protein’s molecular weight to be 70359.61 and to be 641 amino acids long. Positively charged residues (Arg+Lys) and negatively charged residues (Asp+Glu) in the sequence were found to be 83 and 92, respectively. Calculated GRAVY was too low (−0.403) which indicates better interaction of the protein with water, i.e., more hydrophilic nature. The value of the instability index (II) was computed to be 33.03 (<50), therefore, classifying the protein as stable [48]. None of the HSP70’s region was located in transmembrane, as delineated by TMHMM server (Figure-1).

The MSA of 10 different species was investigated to scrutinize the evolutionary relatedness (Figure-2); subsequently, less variation was detected and the major portion of the protein remains conserved among the species under investigation (BB, BI, BT, CH, OA, RN, SS, EC, MM, and HS). The observed sequence variations in HSP70 have been highlighted in white columns and the conserved one in red (Figure-2). The phylogenetic tree for MSA obtained from COBALT server showed BI as evolutionary more closer to BT than BB (Figure-3).

**Figure-2a:**
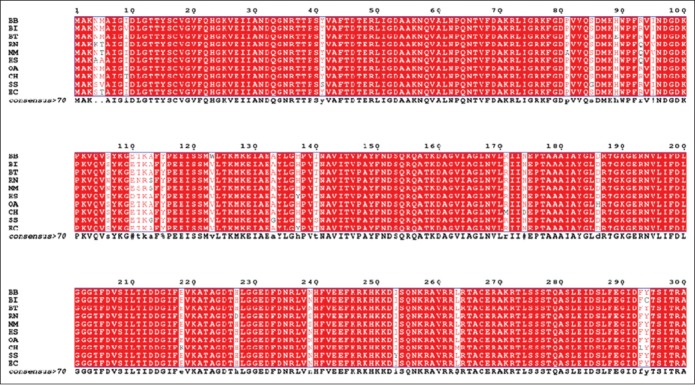
The multiple sequence alignment (1-300 amino acids) for the 10 species. RN=*Rattus norvegicus*, MM=*Mus musculus* SS=*Sus scrofa*, CH=*Capra hircus*, HS=*Homo sapiens*, EC=*Equus caballus*, BI=*Bos indicus*, BB=*Bubalus bubalis*, OA=*Ovis aries*, BT=*Bos taurus*.

**Figure-2b:**
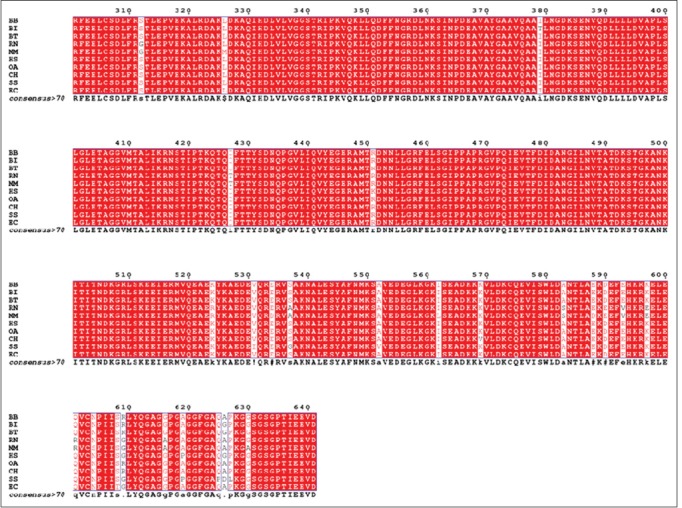
The multiple sequence alignment (301-641amino acids) for the 10 species. RN=*Rattus norvegicus*, MM=*Mus musculus*, SS=*Sus scrofa*, CH=*Capra hircus*, HS=*Homo sapiens*, EC=*Equus caballus*, BI=*Bos indicus*, BB=*Bubalus bubalis*, OA=*Ovis aries*, BT=*Bos taurus*.

**Figure-3:**
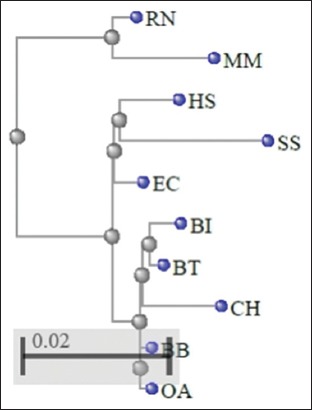
Phylogenetic tree (heat shock protein 70) based on COBALT multiple alignments. BB=*Bubalus bubalis*, BI=*Bos indicus*, BT=*Bos taurus*, CH=*Capra hircus*, OA=*Ovis aries*, RN=*Rattus norvegicus*, SS=*Sus scrofa*, EC=*Equus caballus*, MM=*Mus musculus*, HS=*Homo sapiens*.

Phyre2 module was exploited to deduce the disorder and secondary structure of HSP70 using amino acid sequences. Phyre2 uses the alignment of hidden Markov models through HH search to pointedly improve the precision of alignment and detection rate [49]. A new *ab initio* folding simulation called Poing2 has also been amalgamated to model those regions of the protein which show no homology to known structures [50]. The results showed the highest percentage of alpha-helix region (39%) followed by beta-strand (29%) and the disorder region (13%) was found in the least proportion of the protein.

**Figure-4:**
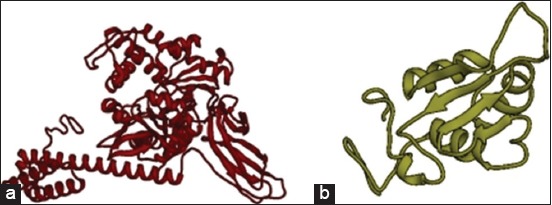
The 3D structure generated for heat shock protein 70 using I-TASSER (a) and heat shock factor-1 using PHYRE2 (b).

**Figure-5:**
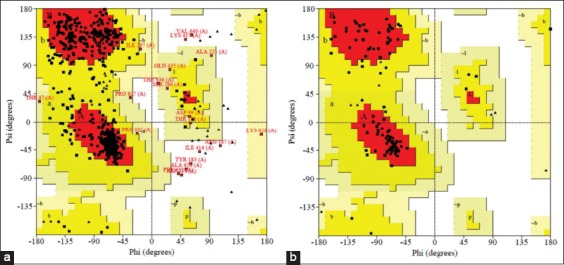
Ramachandran plot statistics of both models analyzed by PROCHECK program of PDBsum [heat shock protein 70 (a) and heat shock factor-1 (b)].

**Figure-6:**
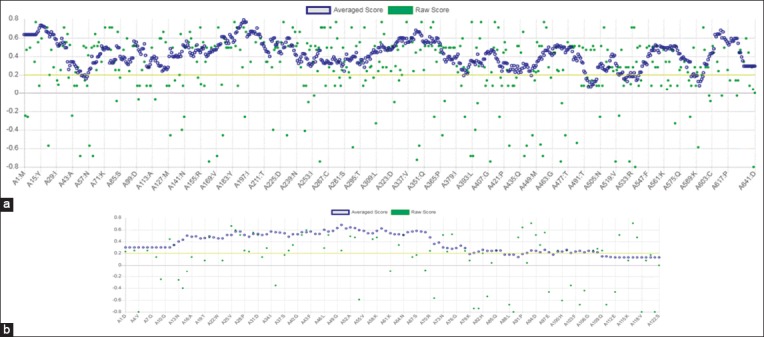
Verifying the 3D model of heat shock protein 70 (a) and heat shock factor-1 (b) by scoring the residues for 3D-1D score.

The three-dimensional (3D) model for HSP70 protein (Figure-4a) and HSF1 (Figure-4b) was first generated by various tools and servers such as Modeller 9.18, RaptorX, Swiss-Modeller, Phyre2, and I-TASSER; the best hit of HSP70 protein was obtained from I-TASSER and the best hit of 3D model of HSF1 generated by Phyre2. I-TASSER can screen out the best 10 templates using the LOMETS threading programs. LOMETS is a meta-server that adopts threading approach comprising numerous threading programs and in each threading program, there can be generated thousands of multiple alignments [51]. I-TASSER only uses the templates which show the highest impact in the threading alignments for building HSP70 model. The Phyre2 uses PDB ID-c2lduA as a template to build HSF1 model, 122 residues have been modeled with 100.0% confidence by the single highest scoring template. As earlier described, HSF1 encompasses 67% disordered region, making it tedious to model the whole protein with a high accuracy rate. In spite of the disorderedness, the Phyre2 modeled the 122 residues of HSF1 with very high accuracy and further refinement of this model was done to obtain precise and refined model of HSF1 along with HSP70. This refinement was done using ModRefiner program since it utilizes an algorithm that uses a two-step strategy for atomic-level energy minimization; through the SPDBV, all computations were done *in vacuo* with the GROMOS96 43B1 parameters set without reaction field. Ramachandran plot statistics of both models shown in Table-2 was analyzed by PROCHECK program of PDBsum (Figure-5a and b). It provides the knowledge of stereochemical features of all protein chains in a given PDB structure [52]. Score “S” gives information about the 3D profile of the amino acid sequences and it should be equal or above the set threshold value, i.e., 80.0%, since the generated 3D protein model showed “S” value more than the threshold value, therefore, considered to be correct. Verify 3D analysis of the best models of HSP70 and HSF1 showed more than 80.0% of the residues having an average 3D-1D score <0.2, indicating that the models were compatible with their sequences (Figure-6a and b). Verify 3D scores are presented in Table-2.

**Table-2:** Quality assessment of HSP70 and HSF1 model.

Quality check	HSP70 (%)	Quality check	HSF1
VERIFY-3D	92.36	VERIFY-3D	82.79
Ramachandran Plot statistics (MFRs, AARs, and GARs)	98.6	Ramachandran plot statistics (MFR, AAR, and GAR)	100.00
Ramachandran Plot statistics (disallowed regions)	1.4	Ramachandran plot statistics (disallowed regions)	0.0

*MFRs=Most favored regions, AARs=Additional allowed regions, GARs=Generously allowed regions

**Figure-7:**
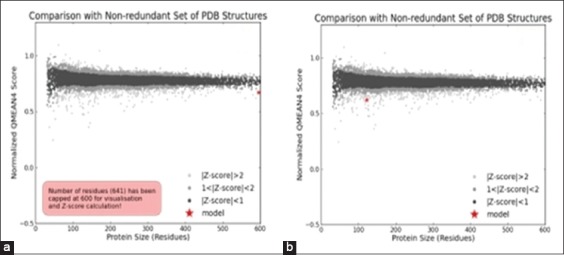
QMEAN value of both models (a: Heat shock protein 70, b: Heat shock factor-1).

As far as concern with Ramachandran plot, more than 98.0% of residues of both the proteins were in the allowed region which, in turn, signifies that the quality of both the models is reliable and good enough because both are as per the acceptance criteria, i.e., at least 90% of residues fall under allowed region. Again, Qualitative Model Energy Analysis termed as QMEAN analysis [53,54] showed the same results, i.e., good quality of models. QMEAN is a composite scoring function narrating the extensive geometrical aspects of protein structures. In this study, values of both the models were found to be around 0.7 which depicts that both models to be having comparable qualities to experimental structures as for a superior quality model QMEAN value must lie between 0 and 1 (Figure-7a and b).

### Protein-protein interactions and docking analysis

**Figure-8:**
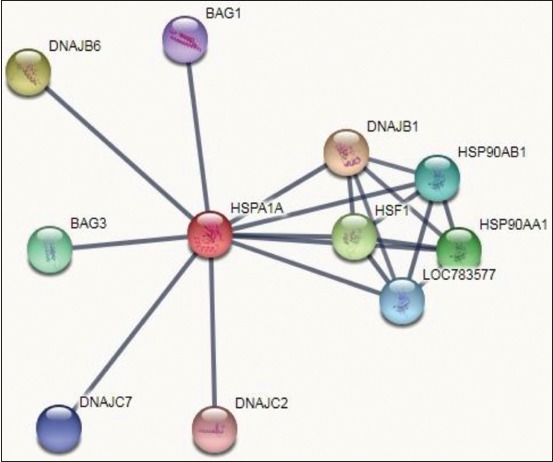
STRING database interaction confidence score of heat shock protein 70/HSPA1A.

A solitary protein may not be able to play each of the committed tasks separately, as a result, these proteins have to be associated with some other proteins or different factor(s) and form channels/pathways or frame buildings that can manage multiple biological events. Similarly, the molecular chaperone(s) requires co-chaperones or some transcription factors, etc., to do their activity efficiently. By the use of STRING database, we are able to know the interacting partners of HSP70. These interacting proteins are mainly molecular chaperones/regulators of molecular chaperones and also found to be participated in signal transduction and cell cycle regulation (Table-3). Interestingly, this database was also enlisted HSF1 which again ensures its importance in heat stress conditions. The STRING database also revealed other proteins that are found to be interacting with HSP70 and are HSP90AA1, HSF1, HSP90AB1, DNAJB1, DNAJB6, BAG3, LOC783577, DNAJC7, BAG1, and DNAJC2. Figure-8 displays the results of STRING database for the interactions with a confidence score of 0.900. The number of nodes, interaction number of edges, and average node degree at this confidence score were 11, 20, and 3.64, respectively.

**Table-3:** HSP70 interacting proteins during cell cycle regulation and signal transduction.

Interacting protein	Protein details	Combined score
HSF1	Heat shock factor protein 1; DNA-binding protein that specifically binds HSEs and activates transcription. In higher eukaryotes, HSF is unable to bind to the HSE unless the cells are heat shocked	0.990
DNAJB1	DnaJ homolog subfamily B member 1; interacts with HSP70 and can stimulate its ATPase activity. Stimulates the association between HSC70 and HIP	0.996
DNAJB6	DnaJ homolog subfamily B member 6; plays an indispensable role in the organization of KRT8/KRT18 filaments. Acts as an endogenous molecular chaperone for neuronal proteins including huntingtin. Suppresses aggregation and toxicity of polyglutamine-containing, aggregation-prone proteins. Has a stimulatory effect on the ATPase activity of HSP70 in a dose-dependent and time-dependent manner and hence acts as a cochaperone of HSP70. Also reduces cellular toxicity and caspase-3 activity	0.994
HSP90AA1	HSP90-alpha; molecular chaperone that promotes the maturation, structural maintenance and proper regulation of specific target proteins involved for instance in cell cycle control and signal transduction. Undergoes a functional cycle that is linked to its ATPase activity. This cycle probably induces conformational changes in the client proteins, thereby causing their activation. Interacts dynamically with various cochaperones that modulate its substrate recognition, ATPase cycle, and chaperone function	0.990
BAG3	BAG family molecular chaperone regulator 3	0.987
HSP90AB1	HSP90-beta	0.986
LOC783577	HSP90-beta	0.985
DNAJC7	Uncharacterized protein	0.982
BAG1	BAG family molecular chaperone regulator 1	0.979
DNAJC2	DnaJ homolog subfamily C member 2; acts both as a chaperone in the cytosol and as a chromatin regulator in the nucleus. When cytosolic, acts as a molecular chaperone: Component of the RAC, a complex involved in folding or maintaining nascent polypeptides in a folding-competent state. In the RAC complex, stimulates the ATPase activity of the ribosome-associated pool of HSP70-type chaperones HSPA14 that bind to the nascent polypeptide chain	0.972

HSP=Heat shock protein, HSE=Heat shock promoter elements, RAC=Ribosome-associated complex

The HSF1 acts as a transcriptional regulator of HSP70; therefore, elucidation of these interactions by docking is very valuable for a general understanding of complex biological mechanisms and pathways. After the quality assessment and model validation step, the modeled ligand protein (HSF1) was docked with receptor (HSP70) homology model using ClusPro 2.0 docking environment. The goal of docking HSP70 and HSF1 was to investigate those docked confirmations, which shows good surface complementarity, which, in turn, can be attained by selecting those complexes that possess the lowest desolvation and electrostatic energies. Only top 10 results out of thousands of synthesized confirmations are revealed by ClusPro 2.0 and the very first model had lowest electrostatic and desolvation potential values (Electrostatic= −824.9, Hydrophobic= −846.6, Van der Waals interaction value = −225.7).

**Figure-9:**
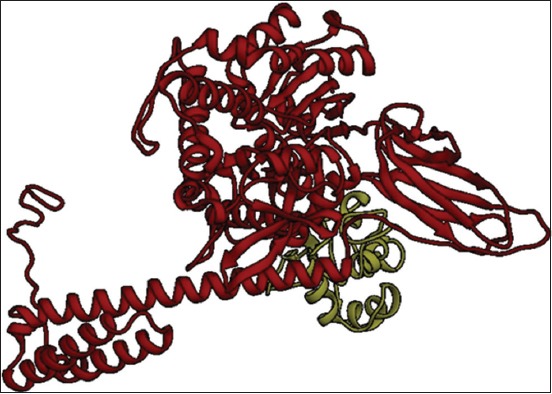
Docked complexes of heat shock protein 70 and heat shock factor-1 as visualized using UCSF Chimera.

**Figure-10:**
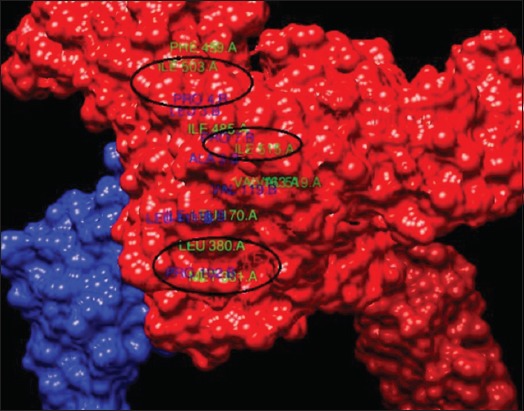
Docked complexes of showing some of the interacting hydrophobic residues.

Final docked complex, HSP70, and HSF1 were analyzed using Protein Interactions Calculator (PIC) web server to confirm the interacting residues [55]. Hydrophobic interactions (within 5 Angstroms), protein-protein main-chain-side-chain hydrogen bonds, protein-protein side-chain-side-chain hydrogen bonds, and ionic interactions were studied. The residues K, R, and E at positions 159, 171, and 523, respectively, of HSP70 showed ionic interactions with residues D, D, and K at positions 114, 114, and 118, respectively, of HSF1. Hydrophobic and all other interactions are given in Tables-4-6. All the structures and docked complexes were finally visualized using UCSF Chimera (Figure-9) and highlighted some of interacting hydrophobic residues (Figure-10).

**Table-4:** Hydrophobic interactions (within 5 Angstroms).

S. No.	Position	Residue	Chain	Position	Residue	Chain
1	163	VAL	A	119	VAL	B
2	170	LEU	A	111	LEU	B
3	170	LEU	A	115	ILE	B
4	380	LEU	A	111	LEU	B
5	381	MET	A	102	PRO	B
6	459	PHE	A	4	PRO	B
7	485	ILE	A	3	LEU	B
8	485	ILE	A	9	ALA	B
9	503	ILE	A	4	PRO	B
10	515	ILE	A	119	VAL	B
11	515	ILE	A	7	PRO	B
12	519	VAL	A	119	VAL	B

**Table-5:** Protein-protein main-chain-side-chain hydrogen bonds.

S. No.	Position	Donor chain	Residue	Atomic	Position	Acceptor chain	Residue	Atomic	Dd-a	Dh-a	MO
1	3	A	LYS	NZ	117	B	ARG	O	2.65	9.99	-
2	159	A	LYS	NZ	8	B	GLY	O	2.75	9.99	-
3	168	A	ASN	OD1	114	B	ASP	O	2.87	1.94	1
4	168	A	ASN	OD1	114	B	ASP	O	2.87	3.71	2
5	168	A	ASN	ND2	114	B	ASP	O	2.86	1.93	1
6	168	A	ASN	ND2	114	B	ASP	O	2.86	3.70	2
7	171	A	ARG	NH1	110	B	GLN	O	2.80	3.70	1
8	171	A	ARG	NH1	110	B	GLN	O	2.80	1.94	2
9	171	A	ARG	NH2	110	B	GLN	O	2.77	3.67	1
10	171	A	ARG	NH2	110	B	GLN	O	2.77	1.90	2
11	487	A	ASN	OD1	2	B	ASP	O	3.01	3.52	1
12	487	A	ASN	OD1	2	B	ASP	O	3.01	1.97	2
13	2	B	ASP	N	487	A	ASN	OD1	3.37	9.99	-
14	8	B	GLY	N	160	A	ASP	OD1	3.09	2.16	-
15	116	B	LYS	N	168	A	ASN	OD1	2.70	1.91	-

Dd-a=Distance between donor and acceptor, Dh-a=Distance between hydrogen and acceptor, MO=Multiple occupancy

**Table-6:** Protein-protein side-chain-side-chain hydrogen bonds.

S. No.	Position	Donor chain	Residue	Atomic	Position	Acceptor chain	Residue	Atomic	Dd-a	Dh-a	MO
1	4	A	ASN	OD1	100	B	GLN	NE2	2.91	2.55	1
2	4	A	ASN	OD1	100	B	GLN	NE2	2.91	2.50	2
3	159	A	LYS	NZ	114	B	ASP	OD1	2.63	9.99	-
4	159	A	LYS	NZ	114	B	ASP	OD2	2.77	9.99	-
5	171	A	ARG	NH1	110	B	GLN	OE1	2.76	2.49	1
6	171	A	ARG	NH1	110	B	GLN	OE1	2.76	2.41	2
7	171	A	ARG	NH2	114	B	ASP	OD2	2.77	1.93	1
8	171	A	ARG	NH2	114	B	ASP	OD2	2.77	2.95	2
9	483	A	ASN	ND2	114	B	ASP	OD2	3.13	2.17	1
10	483	A	ASN	ND2	114	B	ASP	OD2	3.13	3.73	2
11	74	B	ASN	OD1	1	A	MET	SD	3.73	4.38	1
12	74	B	ASN	OD1	1	A	MET	SD	3.73	2.96	2
13	100	B	GLN	NE2	4	A	ASN	OD1	2.91	2.24	1
14	100	B	GLN	NE2	4	A	ASN	OD1	2.91	2.96	2
15	114	B	ASP	OD2	483	A	ASN	ND2	3.13	2.63	1
16	114	B	ASP	OD2	483	A	ASN	ND2	3.13	2.88	2
17	118	B	LYS	NZ	523	A	GLU	OE1	2.62	9.99	-
8	118	B	LYS	NZ	523	A	GLU	OE2	2.56	9.99	-

Dd-a=Distance between donor and acceptor, Dh-a=Distance between hydrogen and acceptor, MO=Multiple occupancy

## Conclusion

This is the first-ever *in silico* interaction study of HSP70 and HSF1 in BB. The investigation has led to the prediction of the secondary structure of HSP70 along with its physicochemical properties study, comparative modeling, protein-protein interaction with HSF1, transcriptional regulation, model evaluation, and its accuracy assessment. The comparative analysis of the HSP70 protein revealed certain significant variations, but most of the sequences were found to be conserved across species. The study delineated a number of amino acid residues of HSP70 potentially interacting with HSF1, which was important to understand the mechanism of heat stress adaptation in BB.

## Authors’ Contributions

RS and RSK designed the experiment. RS and AG wrote the first draft of the manuscript; RS, AG, SKM, SR, AB, and VK performed the *in silico* experimentations. CR and RSK contributed to drafting and revision of the manuscript. All authors read and approved the final manuscript.
